# Design and evaluation of ionically crosslinked multifunctional ELP-SA composite hydrogels for 3D cell culture

**DOI:** 10.1093/rb/rbaf120

**Published:** 2025-11-24

**Authors:** Yiying Chen, Yangmin Wang, Yuxi Li, Xingyang Chen, Wenyun Zheng, Tianwen Wang, Hao Jia, Xingyuan Ma

**Affiliations:** School of Biotechnology and State Key Laboratory of Bioreactor engineering, East China University of Science and Technology, Shanghai 200237, China; School of Biotechnology and State Key Laboratory of Bioreactor engineering, East China University of Science and Technology, Shanghai 200237, China; School of Biotechnology and State Key Laboratory of Bioreactor engineering, East China University of Science and Technology, Shanghai 200237, China; School of Pharmacy, Shanghai Key Laboratory of New Drug Design, East China University of Science and Technology, Shanghai 200237, China; School of Pharmacy, Shanghai Key Laboratory of New Drug Design, East China University of Science and Technology, Shanghai 200237, China; College of Biological and Food Engineering Anhui Polytechnic University, Wuhu 241000, China; Shanghai Key Laboratory for Tumor Microenvironment and Inflammation, Department of Biochemistry & Molecular Cellular Biology, Shanghai Jiaotong University School of Medicine, Shanghai 200025, China; School of Biotechnology and State Key Laboratory of Bioreactor engineering, East China University of Science and Technology, Shanghai 200237, China

**Keywords:** elastin-like polypeptide, sodium alginate, composite hydrogel, 3D cell culture

## Abstract

Hydrogels are 3D crosslinked polymeric networks that can absorb and retain substantial quantities of water or biological fluids. Their soft, hydrated nature and adjustable properties render them highly suitable for a range of biomedical applications, such as drug delivery, tissue engineering and wound healing, by emulating the extracellular matrix. To overcome the limitations associated with the mechanical properties and biological functions of conventional elastin-like polypeptide (ELP) and sodium alginate (SA) hydrogels, a novel ion-responsive two-component ELP-SA hydrogel was developed. ELP variants with functional modules (ELPK/ELPR/ELPS/ELPL) were engineered through genetic techniques and purified to a high degree of purity (>95%) using high-salt-reversible phase-change technology. The release of Ca^2+^ from gluconolactone simultaneously initiated ELP self-assembly and SA ion crosslinking, resulting in the formation of an injectable composite gel within 10 min. This material demonstrated enhanced mechanical properties (storage modulus G′ 450–1773 Pa, pore size 52–103 μm) and reduced swelling (decreased to 60% of that of the SA hydrogel). Functionally, ELPR improved cell adhesion (1.42 times that of collagen I), ELPS facilitated angiogenesis (1.32 times higher than that of the positive control), and ELPL achieved an antibacterial rate exceeding 98% and induced macrophage M2 polarization. This supports the growth of 3D cell spheroids (survival rate of >95%). This modular design synergistically integrates mechanical strength with diverse biological activities, providing an intelligent dressing solution with antibacterial, healing, and anti-inflammatory properties for treating chronic wounds.

## Graphical abstract

**Figure:**
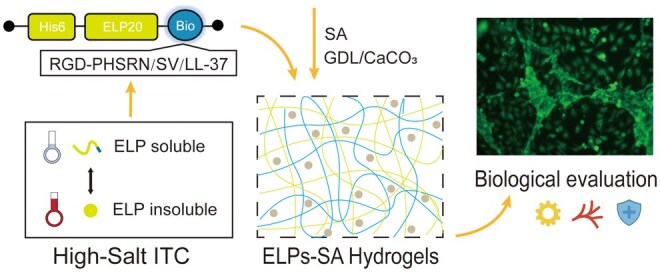

## Introduction

Hydrogels are a type of 3D hydrophilic network characterized by their high water content [1]. They mimic the natural extracellular matrix (ECM) in both structure and hydration properties, making them ideal candidates for biomedical applications where a moist, cell-friendly environment is required [2]. Their excellent biocompatibility, adjustable mechanical properties and biological adaptability have garnered significant interest in various biomedical fields such as wound healing, tissue engineering, drug delivery and 3D cell culture [3]. Nonetheless, creating multifunctional hydrogels that simultaneously exhibit mechanical strength, bioactivity and environmental responsiveness remains a considerable challenge [4].

Elastin-like polypeptides (ELPs) are recombinant protein polymers distinguished by repetitive pentapeptide sequences (VPGXG) derived from native elastin. These sequences emulate the motifs found in tropoelastin, the soluble precursor of elastin, which plays a crucial role in imparting resilience and elasticity to connective tissues [5]. A defining characteristic of ELPs is their reversible phase-transition behavior in response to temperature changes, marked by a lower critical solution temperature [6]. This phase transition is primarily driven by the balance of hydrophobic and hydrophilic interactions within the peptide chain, which can be finely tuned by altering the guest residue or chain length [7]. This property enables self-assembly and phase separation upon heating, facilitating purification through inverse transition cycling (ITC) [8, 9]. The ITC method offers a facile and scalable approach to purifying ELPs without the need for chromatographic techniques, thus reducing cost and processing complexity [10]. The thermoresponsive nature of ELPs enables tunable solubility, drug-loading capacity [11] and environmental responsiveness [12]. These characteristics have positioned ELPs as promising materials in smart drug delivery systems, particularly those requiring on-demand release triggered by physiological stimuli [13]. In addition, ELPs offer genetic programmability, allowing precise control over their amino acid sequences, molecular weights and functional groups [14]. This modularity allows researchers to design ELPs with customized biological functions, such as cell targeting [15], enzymatic activity [16] or immune modulation [17]. However, hydrogels composed solely of ELPs often lack sufficient mechanical strength and typically require covalent crosslinking to form a stable network, which may introduce cytotoxicity or operational complexity [18]. Thus, there is growing interest in combining ELPs with other biomaterials to enhance mechanical performance while retaining their unique biological and thermal responsiveness.

Sodium alginate (SA), a natural anionic polysaccharide, is widely used in biomedical applications owing to its gentle gelation and excellent biocompatibility [19]. When exposed to divalent cations (e.g. Ca^2+^), SA chains undergo ionic crosslinking according to the classical “egg-box” model, resulting in stable hydrogels [20]. However, traditional Ca^2+^-crosslinked SA hydrogels often suffer from rapid gelation and uneven spatial crosslinking during formation, which limits their further application in tissue engineering [21]. To address this issue, a controlled release strategy has been proposed, wherein gluconolactone (GDL) is co-incorporated with calcium carbonate (CaCO_3_). GDL gradually hydrolyzes to release Ca^2+^ ions in a sustained manner, thereby enabling mild and homogeneous ionic crosslinking with SA [22]. Despite the ease of preparation and cell-friendly nature of SA hydrogels, they often display limited mechanical strength and lack the bioactive signals necessary for enhancing cell adhesion [23] or dynamic responsiveness [24]. Therefore, functional modification or synergistic integration with other bioactive components is required to overcome these functional limitations [25, 26].

To harness the advantages of both materials, we propose a strategy for creating multifunctional composite hydrogels through the Ca^2+^-mediated ionic crosslinking of SA and ELPs. In this design, SA provides structural support and ionic responsiveness, whereas ELPs offer thermoresponsiveness and potential bioactivity. This approach aims to overcome the limitations of single-component systems by integrating natural polymers with bioresponsive elements, resulting in a hydrogel platform that is both structurally and functionally synergistic [27].

In this study, the cell adhesion peptide RGD/PHSRN, the human antimicrobial peptide LL-37, and the osteopontin-derived peptide SVVYGLR were introduced at the C terminus of ELPs. Using an *Escherichia coli* expression system, four distinct ELP variants (ELPK, ELPR, ELPS and ELPL) were successfully expressed and purified. Subsequently, composite hydrogels composed of SA and ELP variants (ELP-Vs) were constructed via Ca^2+^-induced crosslinking. The structural characteristics, thermoresponsive behavior and mechanical properties of the hydrogels were systematically evaluated. Furthermore, their biocompatibility *in vitro*, antibacterial activity, regulatory effects on macrophage M2 polarization and pro-angiogenic potential were investigated. This study offers a versatile platform strategy for the development of stimuli-responsive multifunctional hydrogels with extensive applications in tissue engineering and regenerative medicine.

## Materials and methods

### Plasmid construction, protein expression and purification

Initially, all vectors were constructed in *Escherichia coli* DH5α and then transformed into *Escherichia coli* BL21 (DE3) for protein expression. The kZn, RGDSPHSRN, SV and LL37 genes were cloned into the pET-24a vector using BamHI and HindIII restriction sites. The vectors and strains used in this study were provided by BGI (Shenzhen, China). Recombinant plasmids pET24a (+)-ELPI20-KZn, pET24a (+)-ELPI20-RGDSPHSRN, pET24a (+)-ELPI20-LL37 and pET24a (+)-ELPI20-SV were created through T4 ligase-mediated ligation.

### Preparation of starting material

The expression strains were grown overnight in Terrific Bertani medium containing 50 μg/mL kanamycin at 37°C. The overnight cultures were then diluted to a 3% concentration and allowed to grow until the optical density at 600 nm (OD600) reached 0.8–1.0. Induction was carried out with a final concentration of 0.2 mM isopropyl β-D-thiogalactoside (IPTG) at 30°C for 8 h, after which the cells were harvested for further experiments. The ELPK, ELPR and ELPS bacterial pellets were resuspended in equilibration buffer I containing 20 mM phosphate buffer and 500 mM NaCl at pH 7.4. In contrast, the ELPL bacterial pellets were resuspended in equilibration buffer II consisting of 20 mM phosphate buffer, 100 mM NaCl, and 1% glycerin at pH 7.4. In both cases, cell lysis was performed using a high-pressure homogenizer (Union-Biotech, Shanghai, China) at 700 bar and 4°C. The resulting homogenates were centrifuged at 10 000 g for 20 min at 4°C. All the ELP-Vs were detected in the supernatant.

### Immobilized ion metal affinity chromatography

The supernatant of ELP-Vs was filtered through a 0.45-μm sterile filter (Sangon Biotech, Shanghai, China), loaded onto a HisTrap FF Ni^2+^-chelate affinity column (GE Healthcare, UK), and purified using an ÄKTA device (PrimePlus, GE Healthcare, UK). Initially, impurities with weak binding affinities were removed using an equilibrium buffer (20 mM phosphate buffer, 500 mM NaCl, 50 mM imidazole, pH 7.4). The target peptides were subsequently eluted with elution buffer (20 mM phosphate buffer, 500 mM NaCl, and 250 mM imidazole, pH 7.4). The purified peptides were evaluated via sodium dodecyl sulfate-polyacrylamide gel electrophoresis (SDS-PAGE), and protein concentrations were quantified using Bradford’s reagent with bovine serum albumin as the standard. Subsequently, all ELP-Vs were dialyzed against PBS to remove excess ions. Fractions containing the target proteins were concentrated using an Amicon Ultra-15 device (3 kDa MWCO, Millipore, USA) and lyophilized for 24—48 h. Lyophilized proteins were stored at −80°C until further use.

### High-salt precipitation and ITC

#### Thermal behavior of ELP-Vs

The optical density at 350 nm (OD350) of each sample was measured over a range of temperatures using a circular dichroism (CD) spectrometer. The experimental setup included a temperature gradient from 20°C to 80°C in 2°C increments, a heating rate of 1.5°C/min, and wavelength scanning from 347 to 352 nm. The samples consisted of ddH_2_O (baseline), ELPK, ELPR, ELPS and ELPL solutions, each analyzed in triplicate. The raw data were exported as Excel files and the OD350 values at 350 nm were extracted. Data analysis was performed using GraphPad Prism 8, with temperature plotted on the *x*-axis and smoothed curves generated to illustrate temperature-dependent OD350 profiles. The phase-transition temperature (Tt) was defined as the temperature at which the OD350 value first exceeded the baseline [28].

#### Ammonium sulfate precipitation-ITC

Ammonium sulfate (Macklin, Shanghai, China) was gradually added to the lysate supernatant until it reached a final concentration of 20% (w/v) with continuous stirring in an ice-water bath for 10 min. The mixture was then centrifuged at 9000 g for 20 min at 4°C. The supernatant was discarded, and the primary precipitate was resuspended in pre-cooled PBS (4°C) at a volume equal to one-fifth of the initial lysate. The suspension was vortexed until fully dissolved and then centrifuged again under the same conditions (9000 g, 20 min, 4°C) to remove insoluble impurities. The clarified supernatant was transferred to a constant-temperature water bath and subjected to a 10-min thermal shock at the Tt, followed by centrifugation at 9000 g for 20 min at room temperature to collect the thermally aggregated precipitate. For final purification, the precipitate was resuspended in pre-cooled PBS (4°C) at half the initial resuspension volume, centrifuged (9000 g, 20 min, 4°C), and the resulting supernatant containing high-purity ELP-Vs was collected and stored at −80°C for subsequent use.

#### Salt precipitation-ITC

NaCl (Macklin, Shanghai, China) was added to the lysate supernatant to a final concentration of 1 M, followed by thorough mixing. The resulting solution was promptly placed in a constant-temperature water bath for a 10-min thermal shock at Tt. The mixture was then centrifuged at 9000 g for 20 min at room temperature. The supernatant was discarded, and the precipitate was resuspended in pre-cooled ddH_2_O (4°C) at a volume equal to one-fifth of the initial lysate. After centrifuging to ensure complete dissolution, the suspension was centrifuged again (9000 g, 20 min, 4°C), and the supernatant containing purified ELP-Vs was collected and stored at −80°C.

### Far-UV CD analysis of protein secondary structure

Purified protein samples (1.0 mg/mL were dissolved in phosphate-buffered saline). Far-ultraviolet CD spectra were recorded using a quartz cuvette with a path length of 0.1 cm, under a nitrogen purge. Measurements were taken under two distinct temperature conditions: (i) isothermal conditions at 20°C and (ii) a thermal cycling protocol from 20°C to 80°C and back to 20°C. The spectral scanning range was set between 190 and 250 nm, with instrument parameters configured to a bandwidth of 1 nm and a response time of 1 s. Three cumulative scans were averaged for each sample, and baseline corrections were applied using the matched buffer. All measurements were performed in triplicate, and the data were analyzed using GraphPad Prism 8 to visualize temperature-dependent structural transitions.

### Preparation and characterization of ELP-SA gel

Using a synchronous crosslinking strategy, 10 ml of the total volume of the gel precursor solution was used as a representative example. An appropriate quantity of GDL (Sigma-Aldrich, Shanghai, China) was dissolved in 5 mL of 2% w/v SA (Macklin, Shanghai, China) solution using a vortex mixer to ensure thorough dissolution within 1 min. Subsequently, 4 mL of the ELP-V peptide solution at varying concentrations was added and mixed thoroughly. Finally, 1 mL of 0.1 M CaCO_3_ (Sigma-Aldrich, Shanghai, China) solution was added and stirred vigorously for 1 min to achieve a homogeneous precursor. The mixture was then injected into a well plate or mold to facilitate the gradual activation of the ion crosslinking mechanism at room temperature.

#### Optical testing

The hydrogel precursor (200 μl) was added to a 96-well plate. Following the curing process, transmittance was assessed at 590 nm, with air serving as the blank baseline. Each experimental group consisted of three replicates.

#### Rheological testing

Dynamic oscillatory frequency sweep tests were performed on the hydrogels to evaluate their stability under external forces. The gel precursor solution was cast into 12-well plates, forming discs with a diameter of 20 mm and a thickness of 1.0 ± 0.05 mm. After curing, the hydrogels were equilibrated with ddH_2_O at 37°C for 24 h. Subsequently, the samples were placed on a rotating rheometer stage, and the frequency-dependent curves of the storage modulus (G′) and loss modulus (G′′) were recorded under a constant strain of 1% across a frequency range of 0—100 rad/s (20 data points) at 37°C.

#### Scanning electron microscopy analysis

The samples were fractured in liquid nitrogen, freeze-dried to remove moisture, and mounted on a stage using conductive adhesive tape. A gold sputter coating was applied to the samples. The hydrogel microstructure was examined using S-3400N scanning electron microscopy (Hitachi, Japan) at an accelerating voltage of 15 kV.

#### Swelling ratio test

The initial weight of the hydrogel (*W*_0_) was determined. The hydrogel was then fully immersed in 37°C ddH_2_O. At 24, 48 and 72 h, the hydrogel samples were removed, and the blot surface tension-retained solution was removed with filter paper. The swollen wet weight (*W*_S_) was measured, and the swelling ratio was calculated according to Formula (1).


(1) $$\text{Swelling}\;\text{ratio}\left(\%\right)=\left(W_{S}-W_{0}\right)/W_{0}\times 100\%$$

### Cell culture

Michigan Cancer Foundation (MCF-7), human umbilical vein endothelial cells (HUVECs) and mouse monocytic macrophage leukemia cell line (RAW264.7) cells were obtained from the Cell Bank of the Chinese Academy of Sciences (Shanghai, China). The cells were cultured in Dulbecco’s modified Eagle’s medium (DMEM, Gibco, USA) supplemented with 10% v/v FBS (TransGen, China) and 1% v/v penicillin-streptomycin solution (NCM, China) and maintained in a 5% CO_2_ atmosphere at 37°C. Cells (1 × 10^4^ cells/well) were seeded on or inside 96-well glass-bottom plates (NEST, China) with an ELP-SA hydrogel or phenol-red-free Matrigel, respectively. The cell proliferation at 7 days was observed by a fluorescent inverted microscope (IX51, Olympus, Japan).

### Biocompatibility testing of ELP-Vs and hydrogels

#### MTT assay

The biocompatibility of the hydrogels was assessed using the MTT assay (3-(4,5-dimethylthiazol-2-yl)-2,5-diphenyltetrazolium bromide) to evaluate the impact of DMEM solutions containing ELP-Vs and extracts of ELP-SA hydrogels on the proliferation of HUVECs. HUVECs were seeded into 96-well plates at a density of 1 × 10^4^ cells per well and cultured for 24 h under standard conditions (37°C, 5% CO_2_, and 90% humidity). The culture medium was subsequently replaced with the test samples, either the ELP-Vs DMEM solution or ELP-SA hydrogel extract. Following incubation for specified time intervals, 10 μL MTT solution (5 mg/mL) was added to each well and incubated at 37°C for 3 h. The medium was then removed, and 150 μL of DMSO was added to each well. The 96-well plate was agitated for 10 min to ensure the complete dissolution of the formazan crystals. Absorbance was measured at 570 nm using a microplate reader.

#### Calcein-AM/propidium iodide live-dead staining assay

HUVECs were seeded at a density of 5 × 10^5^ cells per well, and their morphology was periodically assessed using an optical microscope. Once the cells adhered and achieved the desired confluence, the original culture medium was removed. Calcein-AM/propidium iodide staining solution (2 μM Calcein-AM/2 μM PI) was applied to completely cover the cells, followed by incubation in the dark at 37°C for 15–30 min. After incubation, the staining solution was discarded. 2D fluorescence imaging was performed using an inverted fluorescence microscope. Calcein-AM, which emits green fluorescence (Ex/Em = 494/517 nm), labeled live cells, whereas PI, which emits red fluorescence (Ex/Em = 535/617 nm), identified dead cells. Z-stack scanning was performed using consistent imaging parameters, with a step size of 3 μm. The images obtained were reconstructed into a 3D fluorescence visualization of the cell culture using the ImageJ software.

### Cell adhesion

The test groups included ELP-Vs (10 μM) and SA (1% w/v), with collagen I (10 μM) as the positive control and PBS-coated wells as the negative control. Three technical replicates were used for each group. First, 24 well plates were coated with the respective solutions (ELP-V, SA, collagen I, or PBS) and incubated at 37°C for 16 h. Prior to the assay, cells were serum-starved for 12 h. After removing the coating solution, the wells were blocked with 200 μl of 1% BSA at 37°C for 1 h, followed by three PBS washes. A single-cell suspension was prepared and seeded into pre-coated wells at a density of 5 × 10^4^ cells/well. The cells were incubated at 37°C for 2–4 h to allow adhesion, after which non-adherent cells were removed by gently washing the wells three times with PBS. The adherent cells were stained with 0.04% crystal violet at room temperature for 15 min, and the stained cells were dissolved in 200 μL of 10% acetic acid. The absorbance was measured at 570 nm (reference wavelength: 630 nm) using a microplate reader. The cell adhesion ratio was calculated using Formula (2).


(2) $$\text{Adhesion}\;\text{rate}\left(\%\right)=\left({\text{OD}}_{\text{experimental}-}{\text{OD}}_{\text{blank}}\right)/\left({\text{OD}}_{\text{positive}\;\text{control}-}{\text{OD}}_{\text{blank}}\right)\times 100\%$$

### Cell wound-healing assay

HUVECs were seeded in 12-well plates at a density of 5 × 10^4^ cells/well. When the cells reached over 90% confluency, the culture medium was replaced with complete medium containing 1% FBS and 1% antibiotics for an additional 12-h incubation. A straight wound was created in the cell monolayer using a 200-μl pipette tip pressed perpendicularly to the well bottom. The wells were gently washed thrice with PBS to remove debris, followed by the addition of serum-free medium containing varying concentrations of ELP-Vs [29]. Each experimental group was tested in triplicate. Cell migration into the wound area was monitored under a microscope at 0, 12, 24 and 36 h post scratching. Images were captured at each time point, and the wound width was quantified using the ImageJ software. The migration rate was calculated using Formula (3).


(3) Migration rate(%)=(1−scratch width at T/scratch width at 0 h)×100%

### *In vitro* angiogenesis assay

Matrigel was evenly coated into 24-well plates and allowed to solidify at 37°C for 30 min. HUVECs were digested with trypsin, centrifuged to remove the supernatant after digestion termination and resuspended in experimental medium (containing 0.38, 0.76, 1.52 or 3.04 μM ELPS) or positive control medium (0.10 μM VEGF). The cell density was adjusted to 2 × 10^5^ cells/mL. Subsequently, 500 μL of the cell suspension was evenly added to the hydrogel surface (final density, 1 × 10^5^ cells/well) and incubated at 37°C in a 5% CO_2_ incubator [30]. Timepoints were selected based on the experimental design (typically 4–24 h), with images captured every 2 h to document the tubulation dynamics. After incubation, the medium was aspirated, and cytoskeletal staining was performed using calcein-AM (live-cell staining). Observations were conducted under an inverted fluorescence microscope (4× or 10× objective lens) with at least five random fields per well (covering the central and peripheral regions). The capillary-like branch number and total capillary network length were quantified using the ImageJ software.

### Determination of antibacterial activity in gel-strain co-culture

A 100-μL hydrogel was prepared in a 1.5-mL EP tube. Subsequently, 100 μL of ATCC 29213 and ATCC 14028 (1 × 10^6^ CFU/mL) were added and co-cultured with the hydrogel at 37°C for 12 h. After incubation, the mixture was diluted 10^6^-fold and spread on LB agar plates. Bacterial colonies were counted after 14 h of incubation, and the antibacterial rate was calculated according to Formula (4).


(4) $$\text{Antibacterial}\;\text{rate}\left(\%\right)=\left(1-{\text{CFU}}_{\text{experimental}}/{\text{CFU}}_{\text{negative}\;\text{control}}\right)\times 100\%$$

### Evaluation of the anti-inflammatory capacity of hydrogels by flow cytometry

Hydrogels (200 μL each of SA, 3.85 μM ELPK-SA and 3.85 μM ELPL-SA) were prepared in 24-well plates and immersed in ddH_2_O at 37°C for 2 h to equilibrate swelling. RAW264.7 cells were seeded onto the hydrogels at a density of 5 × 10^4^ cells/well, with blank wells as controls, and pre-cultured at 37°C in a 5% CO_2_ incubator for 24 h [31]. An equal volume of 155 mM trisodium citrate was added to each well and incubated at 37°C for 10 min to dissolve the hydrogels completely. The cell suspension was gently mixed by pipetting and centrifuged at 500×g for 5 min to collect cells. Cells were washed three times with PBS containing 1% BSA and resuspended in 200 μl of PBS (2 × 10^6^ cells/mL). The suspension was split into two aliquots: one was stained with 5 μL of FITC-CD206 antibody and the other with 5 μL of PE-CD86 antibody, followed by incubation at 4°C for 30 min. The cells were washed three times with PBS containing 1% BSA, resuspended in 200 μL PBS, and filtered through a 40-μm cell strainer. Flow cytometry parameters were as follows: FITC channel: excitation 488 nm, emission 525/40 BP; PE channel: excitation 561 nm, emission 585/42 BP. Raw data were analyzed using FlowJo V10.8 software. The negative threshold was set using the blank group (unstained cells), and the percentages of CD206^+^ and CD86^+^ cells were calculated.

### Statistical analysis

All experiments were conducted with three technical and three biological replicates. Data are expressed as means ± standard deviation (SD). Statistical analysis and graphing were performed using GraphPad Prism software, with one-way analysis of variance (one-way ANOVA) applied to assess significance. Statistical differences were denoted as follows: *P < 0.05 (statistically significant), **P < 0.01 (highly statistically significant), ***P < 0.001 (extremely statistically significant) and “ns” (not significant).

## Results and discussions

### Design of the ELP-Vs

The ELP backbone consists of 20 repeats of the VPGIG pentapeptide sequence, where isoleucine (I) is selected as the guest residue at the X-position to enhance hydrophobicity and lower the Tt, thereby promoting self-assembly under physiological conditions and facilitating subsequent ITC purification. A hydrophilic C-terminal segment, KZn, containing lysine residues for covalent crosslinking and a metal-binding motif, was introduced to endow the ELPs with both covalent and ionic crosslinking capabilities, forming the basic variant ELPK. Furthermore, functional peptides were fused to the C terminus to confer specific bioactivities, including the cell-adhesive peptide RGDSPHSRN (ELPR), pro-angiogenic peptide SVVYGLR (ELPS) and antimicrobial peptide LL-37 (ELPL).

**Figure 1.**
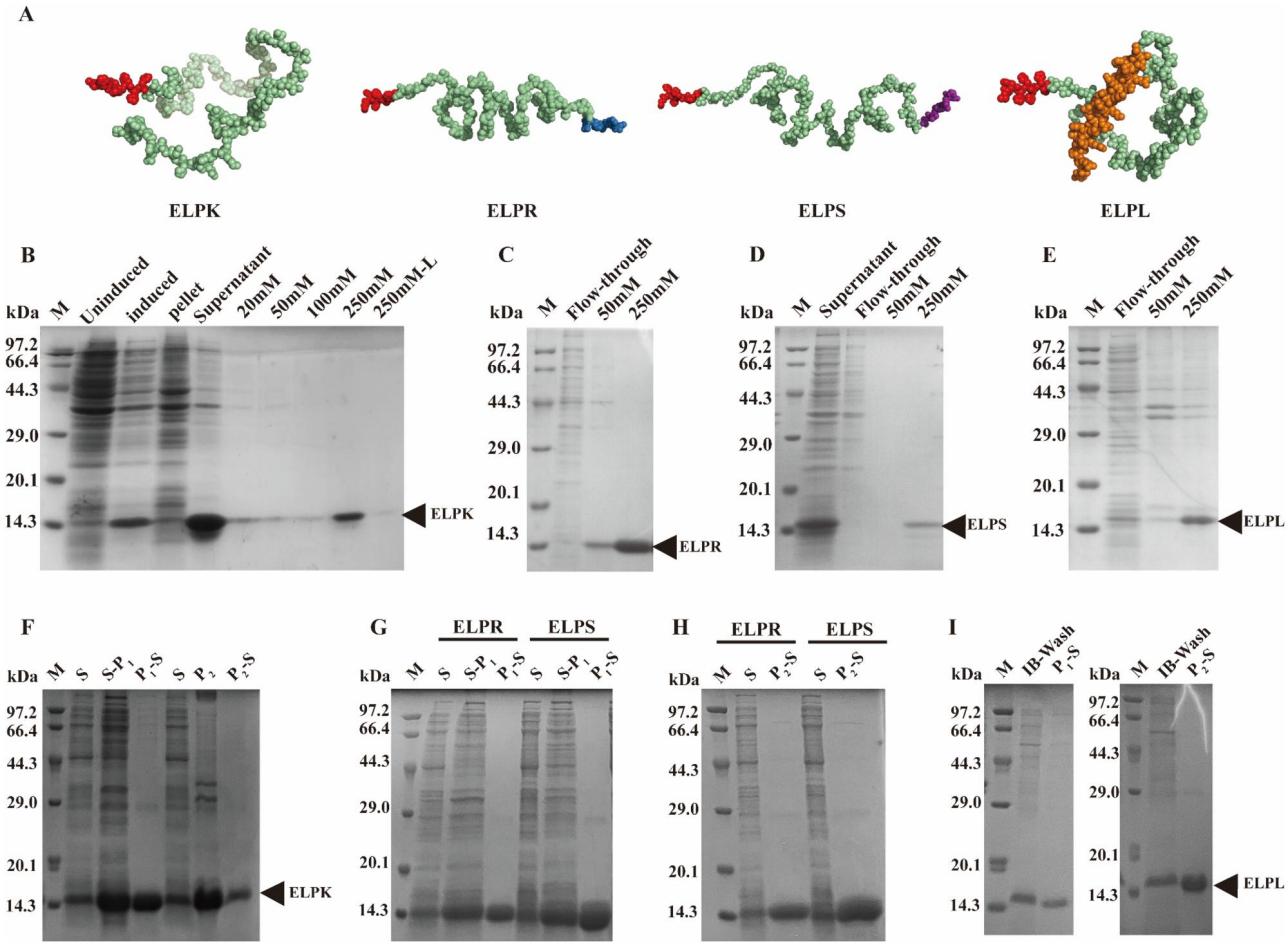
Purification of ELP-Vs expressed in E. coli using Ni^2+^ affinity chromatography and salt-induced ITC. (**A**) Predicted 3D structures of ELP-Vs generated using the AlphaFold2. (Red: His-tag; Green: VPGIG_20_ backbone; Blue: adhesion peptide RGDSPHSRN; Purple: pro-angiogenic peptide SV; Yellow: antimicrobial peptide LL-37.) (**B**) Ni^2+^ affinity chromatography purification of ELPK. (**C**) Ni^2+^-affinity chromatography purification of ELPR. (**D**) Ni^2+^-affinity chromatography purification of ELPS. (**E**) Ni^2+^-affinity chromatography purification of ELPL. (**F**) ITC purification results for ELPK using the two different salt systems. (**G**) ITC purification of ELPR and ELPS using (NH_4_)_2_SO_4_-ITC. (**H**) ITC purification of ELPR and ELPS using NaCl-ITC. (**I**) ITC purification results for ELPL using two salt systems. P_1_-S, supernatant collected after cold centrifugation in PBS following thermal induction at Tt; P_2_, precipitate collected after NaCl-induced phase separation followed by thermal triggering at Tt; P_2_-S, supernatant obtained after dissolving the NaCl-induced precipitate in ddH_2_O; S, supernatant of the cell lysate; S-P_1_, supernatant collected after dissolving the 20% (NH_4_)_2_SO_4_-induced precipitate in PBS and centrifugation.

ProtParam predictions showed that all four ELP-Vs exhibited high hydrophobicity (GRAVY > 0), which was attributed to their uniform VPGIG backbones. However, the fused functional peptides contain multiple charged residues, which help to maintain the overall water solubility of the constructs. As shown in Figure 1A, AlphaFold2-predicted 3D structures of the ELP-Vs indicated that the His-tag was consistently located at the N terminus and was fully exposed without significant steric hindrance, favoring efficient purification via Ni^2+^-affinity chromatography. Variations in the length and charge of the functional segments led to slight differences in the spatial conformation of each variant.

### Expression and purification of ELP-Vs in *E. coli*

In this study, recombinant ELP-Vs were successfully expressed in *Escherichia coli* and efficiently purified from the soluble fraction using an optimized ITC strategy. Protein expression was induced at 30°C, resulting in soluble forms of ELPK, ELPR, and ELPS. Owing to its high charge and aggregation tendency, ELPL requires optimization of the lysis buffer by adding 100 mM NaCl and 1% glycerol to enhance solubility; alternatively, it can be recovered from inclusion body pellets by washing with inclusion body wash buffer.

As shown in Figure 1B–I, ELP-Vs with high purity (87.5–95.7%) were obtained using Ni^2+^-affinity chromatography with 250 mM imidazole elution. By leveraging the unique thermoresponsive properties of ELPs, a sequential ITC purification process was developed based on the order of their Tt (ELPR > ELPK > ELPS > ELPL). This method involves inducing phase separation by adding (NH_4_)_2_SO_4_ or NaCl, followed by re-solubilization in low-ionic-strength PBS or ddH_2_O for purification. Compared to affinity chromatography, the ITC method yielded higher purity (up to 97.5% for ELPL), a shorter process duration (1–2 days), lower cost and required no complex pretreatment (Supplementary Table S1). These results confirmed that ITC is a simple, scalable and highly effective purification strategy suitable for thermoresponsive ELP variants.

### Protein phase-transition properties and secondary structure

**Figure 2.**
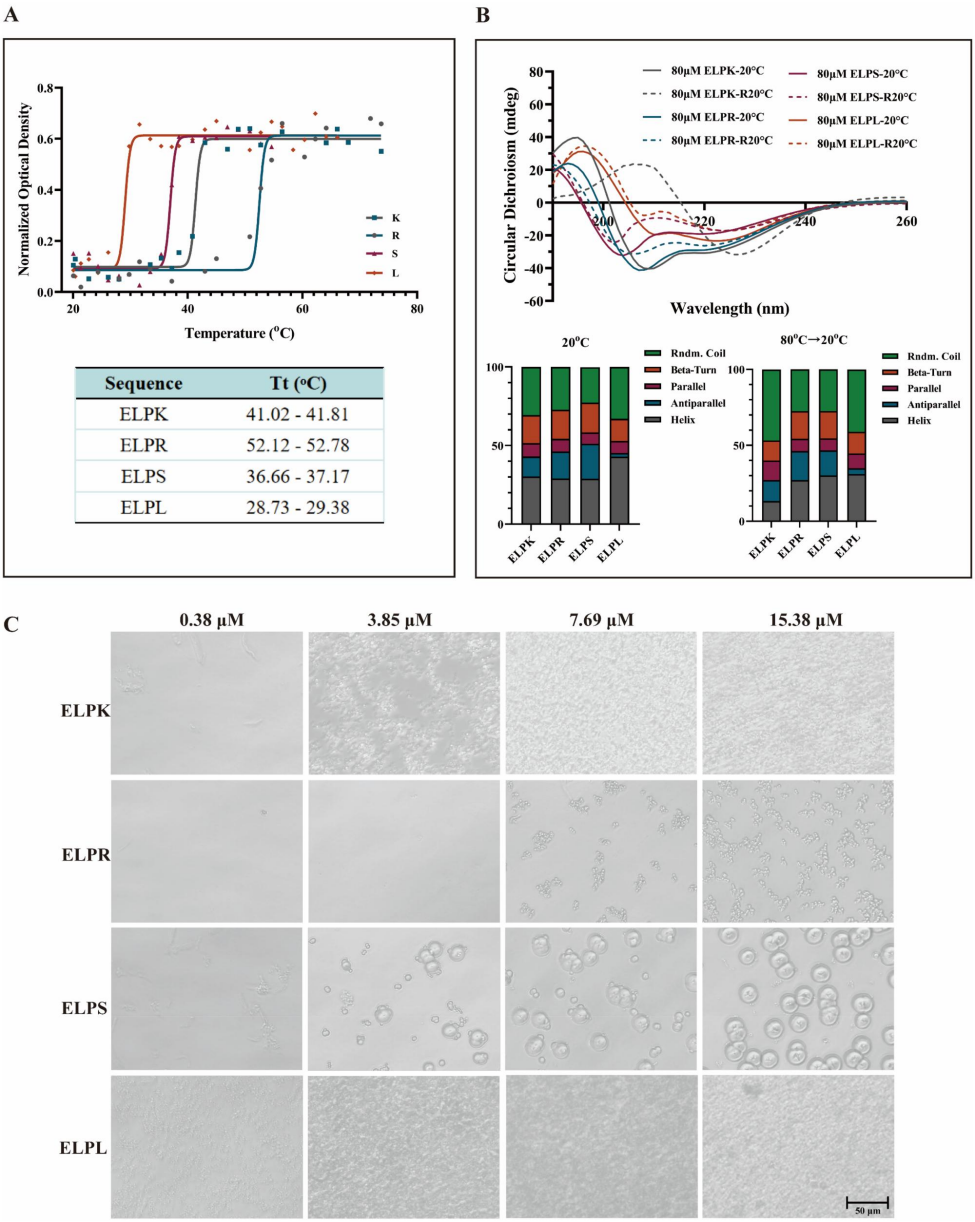
Thermal transition and aggregation behavior of ELP-Vs. (**A**) CD analysis of the transition temperature (Tt) of ELP-Vs. (**B**) CD spectra showing the secondary structure profiles of ELP-Vs. (**C**) Aggregation behavior of ELP-Vs at different concentrations in the complete culture medium at 37 °C.

The basic physicochemical properties of ELPK, ELPR, ELPS and ELPL were analyzed using the ProtParam tool and AlphaFold2 structural prediction. All ELP-Vs were constructed using the highly hydrophobic VPGIG repeat units, and their GRAVY (grand average of hydropathy) values were >0, indicating an overall hydrophobic nature. The temperature-dependent optical transmittance of the ELP-Vs was further measured using UV spectrophotometry (Figure 2A). The results showed a negative correlation between the Tt values and GRAVY scores; that is, the greater the hydrophobicity, the lower the critical Tt, which is consistent with the known thermoresponsive behavior of ELPs [32].

CD spectroscopy revealed that at room temperature, ELP-Vs primarily adopted α-helical and random-coil secondary structures. Upon heating (from 20°C to 80°C and then cooling to 20°C), the characteristic peak at 222 nm gradually decreased, indicating α-helix unfolding and a reversible conformational change, supporting their thermally driven phase-transition behavior (Figure 2B). This temperature-sensitive conformational transition also provides a theoretical basis for ITC purification, where selective precipitation and re-solubilization of ELPs are driven by their phase-transition properties [10].

Further analysis of the particle size distribution at physiological temperature (37°C) revealed distinct self-assembly behaviors among ELP-Vs (Figure 2C). Highly cationic ELPL (fused with the antimicrobial peptide LL-37) readily formed nanoparticles in PBS, whereas negatively charged ELPR and ELPS (fused with RGD and SV peptides, respectively) tended to aggregate into micron-scale particles. These findings indicate that the surface charge state of the protein plays a critical role in modulating its self-assembly behavior, in line with a “hydrophobicity-driven, electrostatically modulated” dual mechanism [33]. This suggests that precise control over the aggregation state and particle size can be achieved by tuning the charge distribution via sequential design.

### Construction and physical properties of ELP-SA hydrogels

**Figure 3.**
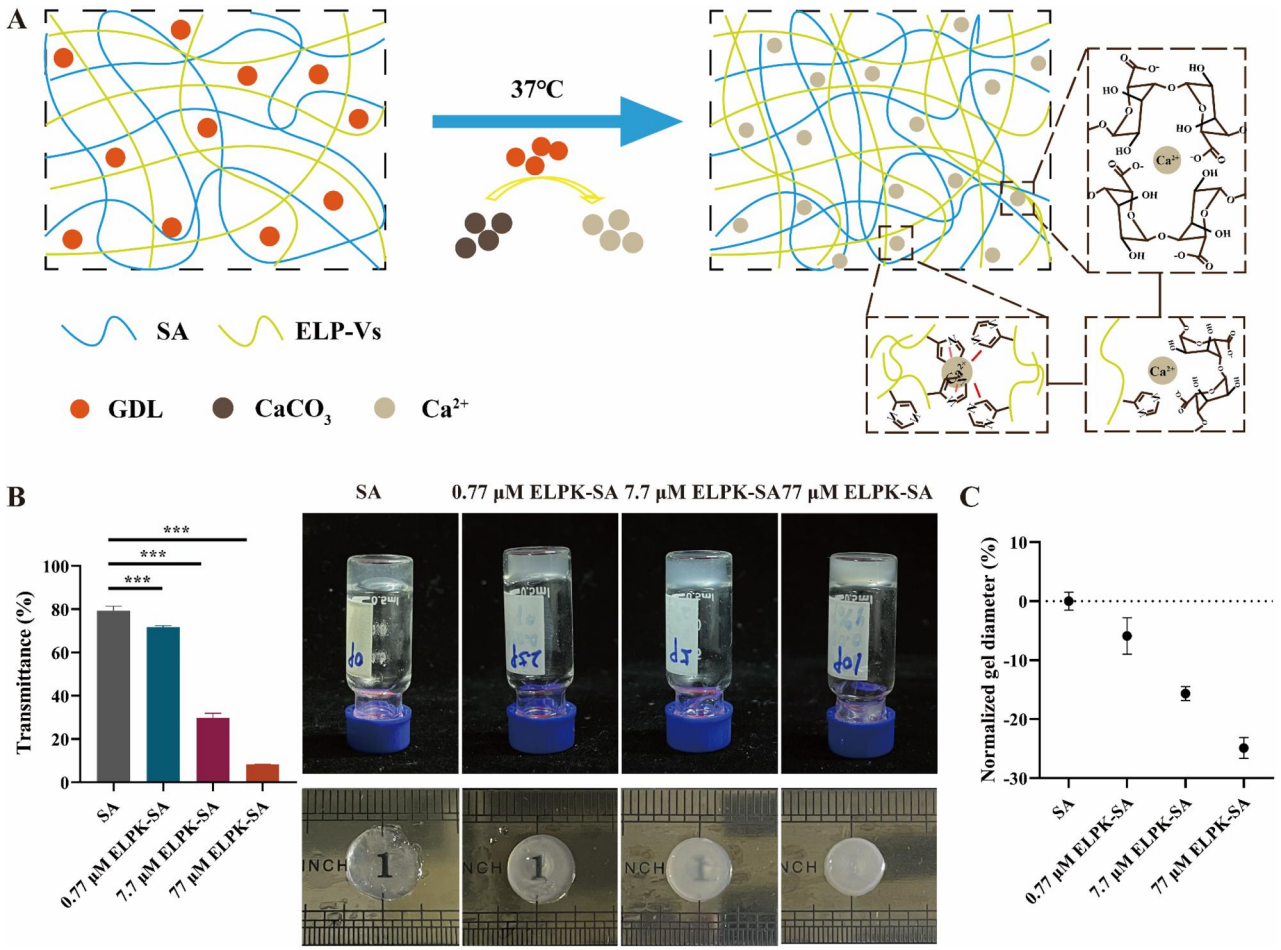
Schematic illustration of the formation and structural features of ELP-SA hydrogels. (**A**) Crosslinking mechanism of the ELP-SA hydrogels. (**B**) Macroscopic transparency of the hydrogels under natural light. (**C**) Transmittance at 590 nm and diameter shrinkage rate of hydrogels at 37°C.

To optimize hydrogel properties, we systematically adjusted key preparation parameters to ensure reproducible gelation and functional performance. First, the effect of the Ca^2+^/GDL molar ratio on gelation was examined: under conditions of 1% (w/v) SA and 10 mM CaCO_3_, GDL was added to achieve molar ratios of 1:0.5, 1:1, 1:2, 1:4 and 1:5. When the Ca^2+^/GDL ratio was set to 1:4, CaCO_3_ fully dissolved with no crystalline residue, and the gelation time was reduced from 60 to 5 min. Second, the effect of Ca^2+^ concentration on gel performance was evaluated while keeping the SA concentration at 1% (w/v) and the CaCO_3_/GDL ratio at 1:4. CaCO_3_ concentrations of 10, 20, 30 and 40 mM were tested, and increasing Ca^2+^ from 10 to 30 mM shortened the gelation time from 15 to 1 min, indicating accelerated ionic crosslinking. An SA/Ca^2+^ ratio of 1% w/v: 10 mM was selected for subsequent experiments, providing a moderate gelation time suitable for injection molding [34]. Third, the incorporation of ELP-Vs (ELPK as a model, 0.77—77 μM) into the hydrogel enabled the construction of composite double-network hydrogels by combining Ca^2+^ ionic crosslinking with ELP hydrophobic self-assembly (Figure 3A). The addition of ELPK further shortened gelation time from 15 to 10 min and significantly enhanced the hydrogel’s mechanical strength. As the ELPK concentration increased, the hydrogel transmittance markedly decreased (as low as 8.21% at 77 μM), indicating increased turbidity due to hydrophobic aggregation. At 37 °C, the gel shrinkage ratio was linearly correlated with the ELPK concentration (*R*^2^ = 0.95), reaching a maximum shrinkage of 24.87%, confirming its robust thermoresponsive behavior (Figure 3B and C).

**Figure 4.**
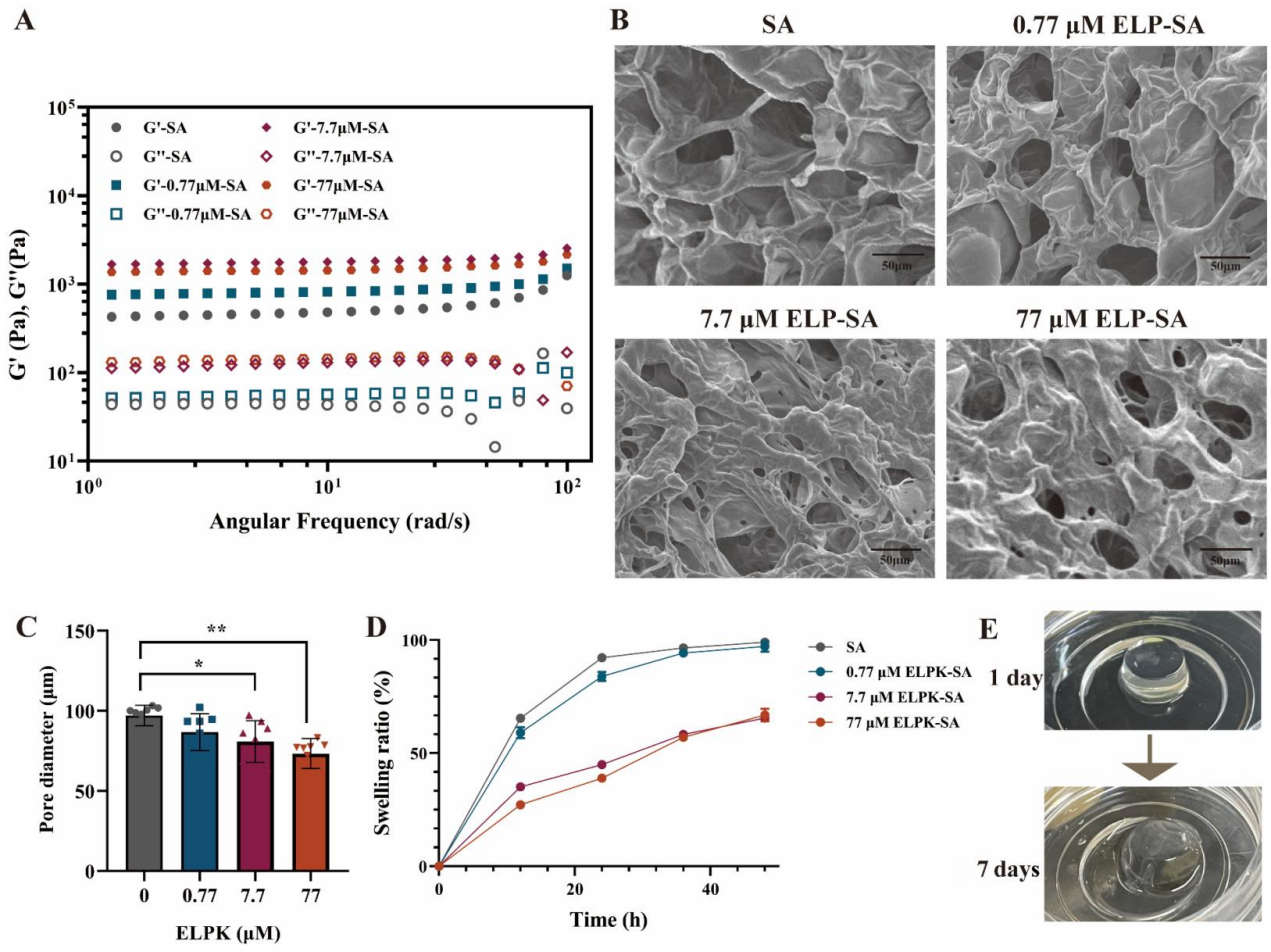
Physicochemical characterization of the ELP-SA hydrogels. (**A**) G′ and G″ of hydrogels measured by dynamic oscillatory frequency sweep. (**B**) SEM images of freeze-dried ELP-SA hydrogels (scale bar = 50 μm). (**C**) Pore size distribution of ELP-SA hydrogels based on SEM analysis. (**D**) Swelling ratio of ELP-SA hydrogels over time at identical ELP concentrations. (**E**) Morphological changes of the 0.77 μM ELP-SA hydrogel during degradation (‘*’ indicates P < 0.05, a statistically significant difference; ‘**’ indicates P < 0.01, a highly significant difference.).

Dynamic rheological measurements showed that the G′ of the ELPK-SA composite hydrogels was significantly higher than that of SA alone (478.9 Pa), reaching a peak of 1772.95  Pa (7.7 μM ELPK). However, excessive 77 μM ELPK led to a decline in G′ to 1444 Pa, suggesting that over-crosslinking may induce gel brittleness [35] (Figure 4A). In contrast, the G′ of single-component ELP hydrogels is only around 200 Pa [32], whereas our results demonstrate that the G′ of ELP-SA composite hydrogels can be tuned within the physiologically relevant range (0.1–50 kPa) [36]. SEM revealed that all hydrogels exhibited porous structures, with the average pore size decreasing from 97.0 μm in SA to 73.3 μm in 77 μM ELPK, better matching the size range of mammalian cells (3–30 μm) (Figure 4B and C). The swelling behavior of the hydrogels was also regulated by the ELPK concentration; higher concentrations (7.7–77 μM) suppressed swelling (from 98.8% to 64.5%), likely due to reduced carboxyl group ionization caused by hydrophobic aggregation (Figure 4D). Further studies demonstrated that ELPK significantly delayed the degradation of SA gels in PBS, allowing structural stability for up to 7 days. This observation aligns with the degradation behavior of SA hydrogels reported in the literature [37]. Additionally, adjusting the ionic strength, such as chelating Ca^2+^ with citrate, can achieve rapid degradation within 10 min. This is due to the competitive chelation of Ca^2+^ by citrate ions, which leads to the collapse of the ionic crosslinking network. Based on the above comparative analysis, the degradation of ELP-SA hydrogels in a buffer with a certain ionic strength occurs because anions competitively chelate Ca^2+^, causing the gradual removal of calcium ions from the gel network and resulting in structural collapse [38]. Hydrogels containing higher concentrations of ELPK degrade more slowly, likely because thermoresponsive aggregation forms a more hydrophobic surface, slowing solvent exchange and thereby reducing the rate of Ca^2+^ loss, which prolongs the degradation time (Figure 4E).

### Cytotoxicity and proliferative effects of ELP-Vs on HUVECs and biocompatibility of ELP-SA hydrogel

**Figure 5.**
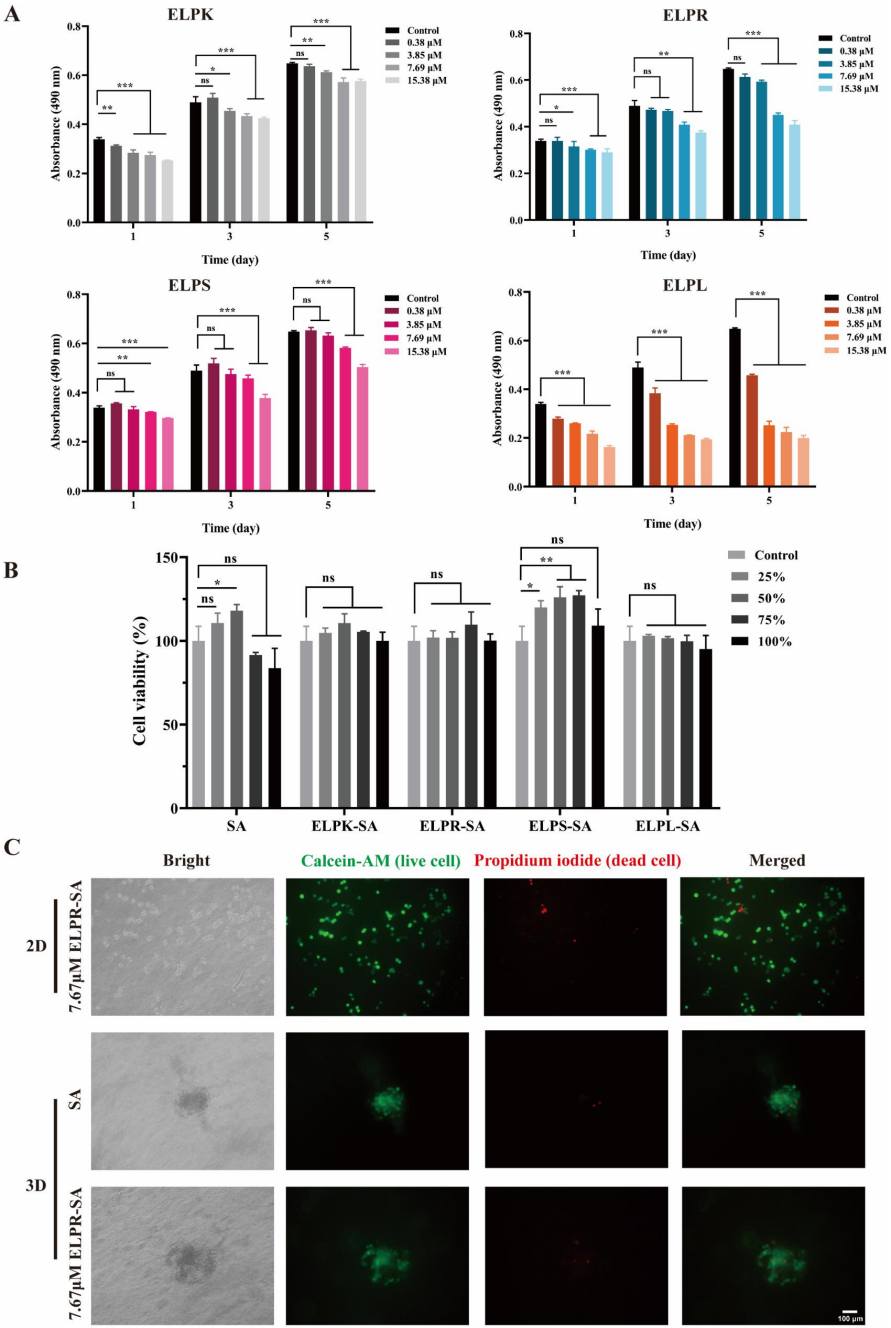
Cytocompatibility evaluation of ELP-Vs and ELP-SA hydrogels. (**A**) Viability of HUVECs treated with different concentrations of ELP-Vs at various time points, assessed by MTT assay. (**B**) Viability of HUVECs after 24-h incubation with extracts from ELP-SA hydrogels, measured by MTT assay. (**C**) Live/dead staining of HUVECs after treatment with hydrogel extracts using Calcein-AM/PI (scale bar = 100 μm; ‘*‘ indicates P < 0.05, a statistically significant difference; ‘**’ indicates P < 0.01, a highly significant difference; ‘***’ indicates P < 0.001, an extremely significant difference; ‘ns’ indicates no significant difference.).

The effects of different concentrations of ELP-Vs (ELPK, ELPR, ELPS and ELPL) on HUVECs viability were assessed using an MTT assay. As shown in Figure 5A, after 1, 3 and 5 days of treatment with ELPK or ELPS at concentrations ranging from 0.38 to 15.38 μM, the cell viability remained above 70%, meeting the ISO 10993-5 criteria for non-cytotoxicity. In contrast, ELPR and ELPL exhibited potential cytotoxicity at higher concentrations: at 15.38 μM, ELPR reduced cell viability to 50–70% on day 5, whereas ELPL caused cell viability to drop below 50% within the 3.85–15.38 μM range. This phenomenon may be attributed to the hydrophobic aggregation of ELPs at physiological temperatures, which could limit nutrient diffusion in the culture medium and the inherent cationic cytotoxicity of the LL-37 peptide [39]. Based on these findings, subsequent experiments were conducted with ELP-V concentrations of ≤3.85 μM.

The biocompatibility of the ELP-SA composite hydrogel was evaluated. As shown in Figure 5B, HUVECs treated for 24 h with various concentrations (25–100%) of hydrogel extracts containing 7.69 μM ELPs exhibited cell viability above 70%. Notably, the extract from the ELPL-SA hydrogel resulted in a cell viability of 95.09%, which was significantly higher than that observed in the free ELPL solution group (61.81%), indicating that hydrogel encapsulation effectively mitigated its cytotoxicity. As shown in Figure 5C, calcein-AM/PI staining further confirmed the excellent cytocompatibility of ELPR-SA hydrogels, with a live-cell rate of up to 94.92%. Although ELPR contains the RGD motif known to enhance cell adhesion, its spatial accessibility may be limited after immobilization, suggesting that future studies should optimize the exposure or density of functional peptides to improve cell-binding efficiency.

### Adhesive function of ELPR

Studies have shown that the RGD peptide (Arg-Gly-Asp) and the PHSRN sequence (Pro-His-Ser-Arg-Asn) serve as binding sites for αvβ3 integrin, which not only mediates physical cell adhesion but also regulates key cellular activities such as migration, proliferation, and survival through dynamic coupling of the ECM with cytoskeletal remodeling. Functionalization with RGD/PHSRN has thus become an important strategy in biomaterial design [40].

**Figure 6.**
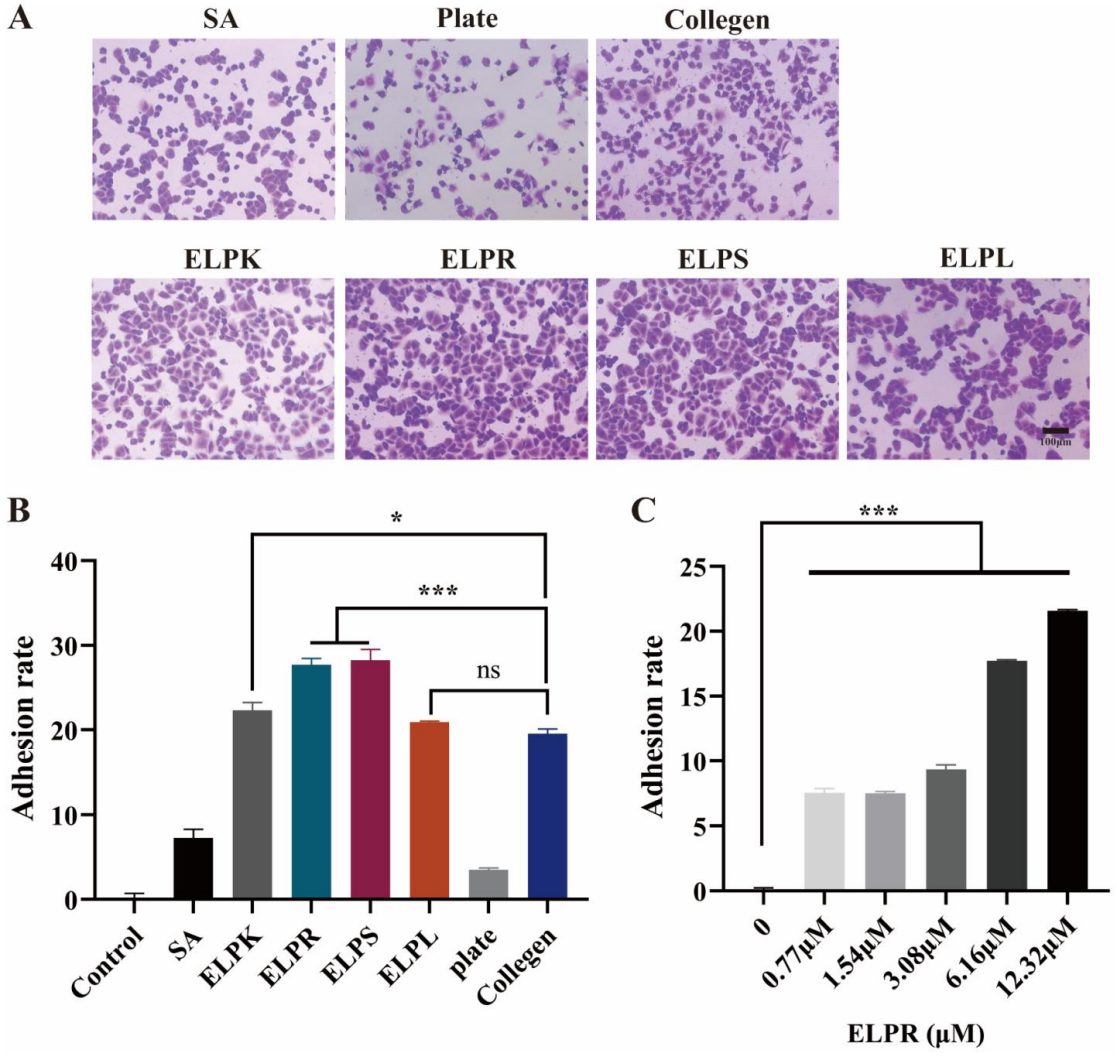
Effect of ELP-Vs coating on HUVEC adhesion. (**A**) Morphology of HUVECs that adhered to plates coated with different ELP-Vs after 2 h of incubation (scale bar = 100 μm). (**B**) Quantification of HUVEC adhesion to different ELP-V-coated plates within 2 h. (**C**) Adhesion rate of HUVECs on ELPR-coated plates with gradient concentrations after 2-h incubation (‘*’ indicates P < 0.05, a statistically significant difference; ‘***’ indicates P < 0.001, an extremely significant difference.).

In this study, these adhesion sequences were fused to the C terminus of the ELP repeat sequence, aiming to enhance the cell-adhesive properties of the hydrogel material. As shown in Figure 6A and B, HUVEC adhesion in the ELPR and ELPS groups was 1.42-fold and 1.44-fold higher, respectively, than that in the collagen I positive control. Among them, ELPR primarily promoted adhesion through direct integrin recognition, whereas ELPS may indirectly enhance adhesion and spreading by activating pro-angiogenic signaling pathways.

Further concentration-dependent analysis (Figure 6C) showed that within the 0.77–13.32 μM range, cell adhesion treated with ELPR increased continuously with concentration, without reaching a clear saturation plateau. This indicates that ELPR exhibits strong and tunable adhesion capacity within the experimental concentration range. This effect may be attributed to the conformational stability of the ELP peptide chain, which allows full exposure of the RGD/PHSRN motifs, facilitating integrin binding and thereby enhancing cell anchoring and adhesion signaling [41]. These results suggest that ELPR could provide a controllable adhesive microenvironment for *in vitro* 3D cell culture or tissue engineering scaffolds.

### Enhancement of HUVEC migration and angiogenesis by ELPS

The SV sequence is an important functional motif of osteopontin, possessing multiple functions including regulation of angiogenesis, recruitment and induction of stem cell differentiation, and modulation of the inflammatory microenvironment. Its pro-angiogenic activity is comparable to that of VEGF. Studies have shown that SV-functionalized hydrogels can promote endothelial cell proliferation and accelerate the formation of vascular networks [42]. In this study, the SV sequence was partially fused to the C terminus of the ELP repeat sequence to enhance the pro-angiogenic capability of the hydrogel.

**Figure 7.**
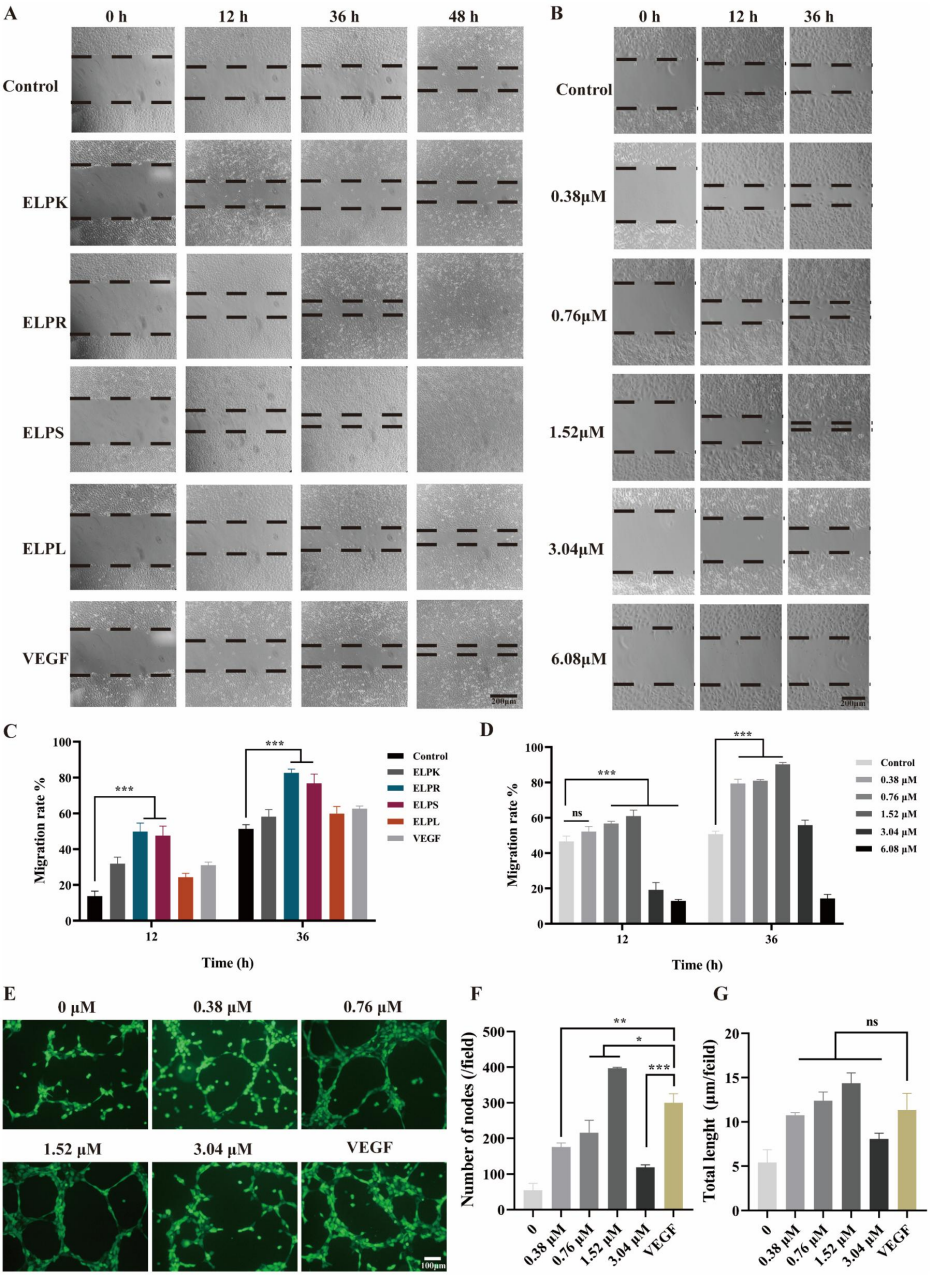
Effects of ELP-Vs on HUVEC migration and angiogenesis. (**A**) Representative images of HUVEC migration after treatment with different ELP-Vs. (**B**) Representative images of HUVEC migration after treatment with various concentrations of ELPS. (**C**) Quantitative analysis of the HUVEC migration rate in response to different ELP-Vs. (**D**) Quantitative analysis of the HUVEC migration rate under varying concentrations of ELPS (scale bar = 200 μm). (**E**) Representative calcein-AM-stained images of tubular structures formed by HUVECs after 12 h (scale bar = 100 μm). (**F**) Quantification of branch points in tube formation using ImageJ software. (**G**) Quantification of total tube length using ImageJ (‘*’ indicates P < 0.05, a statistically significant difference; ‘**’ indicates P < 0.01, a highly significant difference; ‘***’ indicates P < 0.001, an extremely significant difference; ‘ns’ indicates no significant difference.).

As shown in Figure 7A–D, ELPS exhibited a clear concentration-dependent effect on HUVEC migration and angiogenesis. Treatment with 1.52 μM ELPS for 36 h increased the cell migration rate to 76.73%, which was significantly higher than that of the blank control (51.31%) and positive control VEGF (62.66%). However, at concentrations exceeding 1.52 μM, the migration rate declined markedly. In the tube formation assay (Figure 7E–G), 1.52 μM ELPS induced the formation of 397 branches per field, surpassing the VEGF group (300 branches), although the total vessel length showed no significant difference, indicating that ELPS primarily enhances branching complexity rather than overall vascular extension. Similarly, higher concentrations of ELPS did not further promote angiogenesis, likely because of peptide aggregation and sedimentation at high concentrations, resulting in a reduction in the effective bioavailable dose. The findings indicate that the recombinant ELPS effectively promotes endothelial cell migration, defining a key concentration threshold for its functional application. Moreover, hydrogel-based sustained delivery may preserve effective local concentrations of ELPS, representing a promising approach to potentiate its sustained migration-enhancing activity *in vitro* or in tissue engineering contexts [43].

### Antibacterial and anti-inflammatory activities of ELPL

LL-37 is a human-derived cationic antimicrobial peptide composed of 37 amino acids, broadly expressed in epithelial and immune cells [44]. Due to its broad-spectrum antibiofilm activity and wound-healing promotion, LL-37 is advantageous for the repair of chronically infected wounds and is commonly applied in antimicrobial dressings, hydrogels, and tissue engineering scaffolds to improve the local immune microenvironment and accelerate tissue regeneration [39, 45]. In this study, LL-37 was fused to the C terminus of the ELP repeat sequence to enhance the hydrogel’s antibacterial properties.

**Figure 8.**
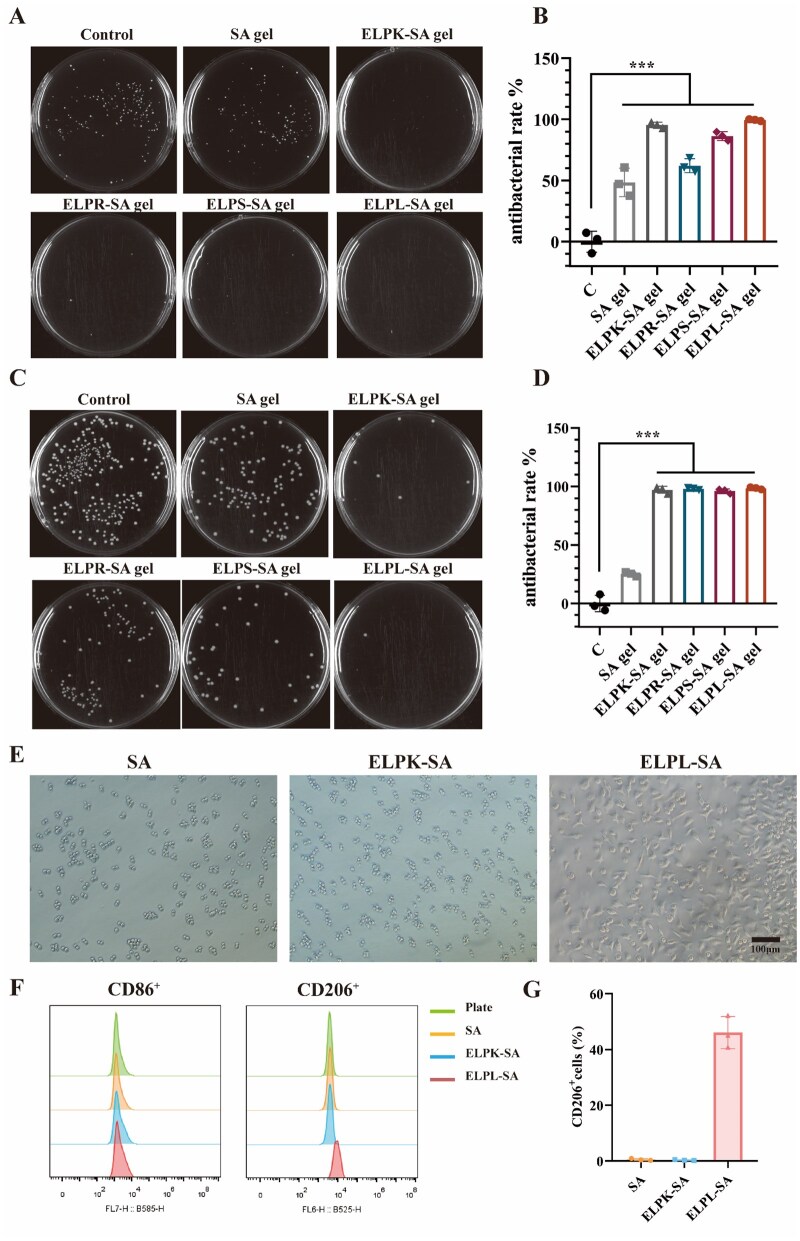
Antibacterial activity and immunomodulatory effects of ELP-SA hydrogels. (**A**) Colony formation assay of Staphylococcus aureus after 12 h of co-culture with ELP-SA hydrogels. (**B**) Quantitative analysis of the *S. aureus* inhibition rate after 12 h of co-culture with ELP-SA hydrogels. (**C**) Colony formation assay of *Salmonella* spp. after 12 h of co-culture with ELP-SA hydrogels. (**D**) Quantitative analysis of Salmonella inhibition rate after 12 h of co-culture with ELP-SA hydrogels. (**E**) Morphology of RAW 264.7 macrophages after 36 h co-culture with hydrogels. (**F**) Flow cytometry analysis of macrophage phenotypes after 36 h of co-incubation. (**G**) Quantification of M2 polarization based on the proportion of CD206^+^ macrophages (‘***’ indicates P < 0.001, an extremely significant difference).

The ELPL-SA hydrogel exhibited potent antibacterial and immunomodulatory properties. In its free form, ELPL exhibited strong antibacterial activity against Staphylococcus aureus (MIC = 1.52–3.04 μM), but showed relatively weaker inhibitory effects against Salmonella (with an inhibition rate of 87.8% at 24.32 μM) (Supplementary Figure S1). As shown in Figure 8A–D, after incorporation into a 7.7-μM ELPL-SA hydrogel, over 95% inhibition was achieved against both bacterial strains. This enhanced efficacy may be attributed to the improved exposure and sustained release of LL-37 within the hydrogel network, which enhanced its bioactivity and stability. Meanwhile, the ELPK-SA hydrogel also exhibited significant antibacterial activity, which may be attributed to the higher number of positive charges at its C terminus, enabling interaction with bacterial membranes and causing membrane disruption [46]. SA, ELPR-SA, and ELPS-SA hydrogels also showed a certain degree of antibacterial effect, likely due to the slightly acidic environment generated during hydrogel preparation (pH = 5.42 ± 0.18) [47].

Previous studies have shown that the *in vivo* antibacterial activity of LL-37 primarily relies on its cationic properties to disrupt microbial membranes, while also promoting macrophage polarization and activating immune responses [48]. Therefore, representative surface markers of M1 and M2 macrophages, CD86 and CD206, were used to evaluate the immunomodulatory effect of the composite hydrogel on macrophage polarization. As shown in Figures 8E–G, treatment of RAW 264.7 macrophages with 7.7 μM ELPL-SA hydrogel for 36 h, significantly promoted macrophage polarization toward the M2 phenotype, with CD206^+^ cells reaching 46.1%. During the co-culture process, it was observed that after 24 h, only 10% of RAW 264.7 cells were polarized. By 36 h, the polarization rate increased sharply, indicating that the ELPL-SA hydrogel in the culture medium effectively released ELPL to induce RAW 264.7 polarization. M2 polarization reflects an anti-inflammatory and tissue-repairing immune response, promoting ECM remodeling and stimulating angiogenesis through growth factor secretion, while simultaneously suppressing excessive inflammation to prevent secondary tissue damage. This characteristic suggests that the material also holds potential for applications in chronic inflammatory diseases and tissue engineering scaffolds [49].

### Functionalized ELP-SA hydrogel for 3D culture of MCF-7 cells

**Figure 9.**
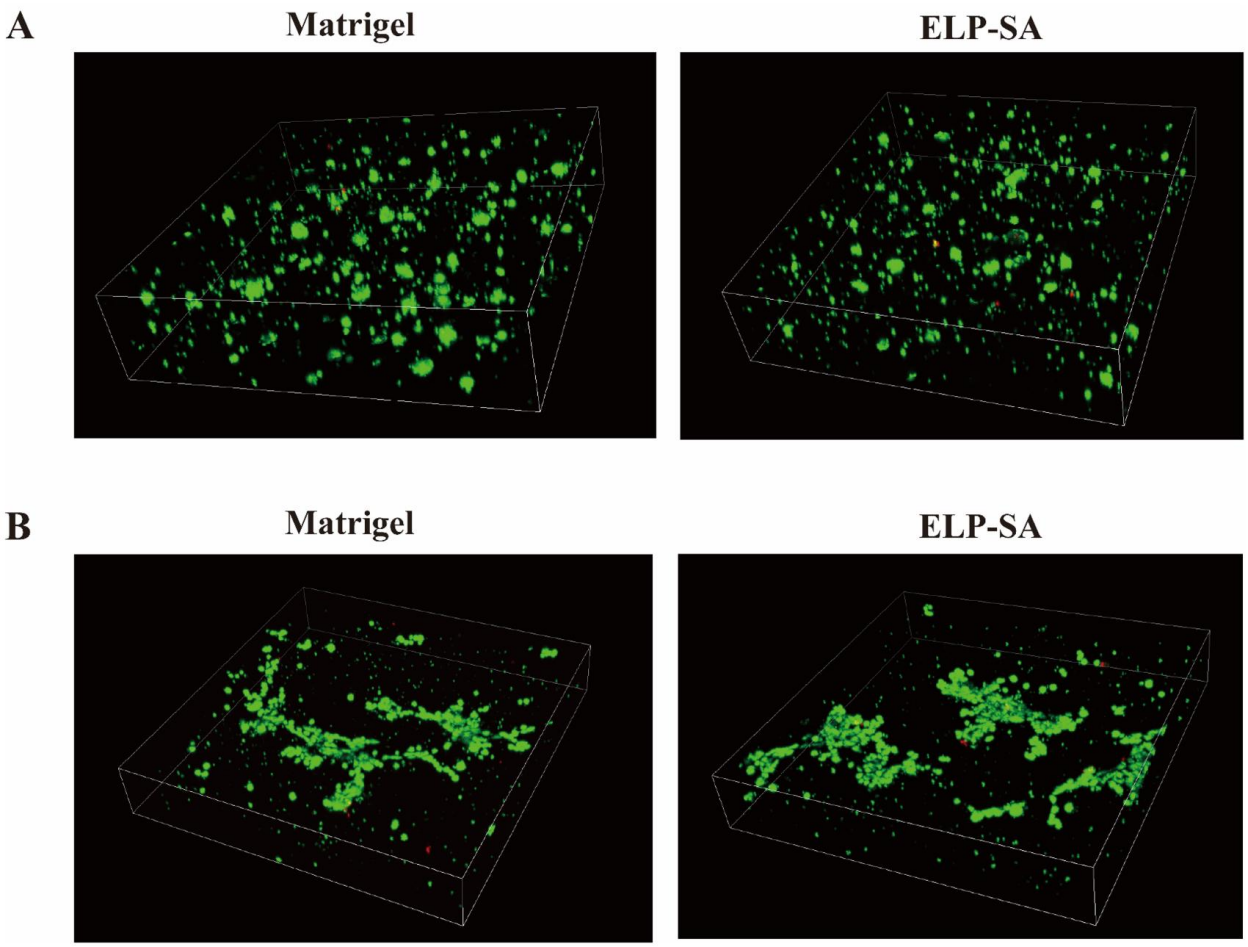
3D Visualization of MCF-7 cell morphology in ELP-Vs-SA hydrogels. (**A**) Representative 3D confocal images of MCF-7 cells cultured in ELP-Vs-SA hydrogels (thickness, 300 μm). (**B**) Representative 2D images of MCF-7 cells in ELP-Vs-SA hydrogels (thickness = 200 μm; green: calcein-AM, live cells; red: PI, dead cells).

The composite hydrogel (77 μM ELPR + 3.04 μM ELPS + 1% SA) effectively supported stable 3D culture of MCF-7 cells (Figure 9). After five days of culture, cell viability exceeded 95%, and dense spheroids with diameters ranging from 50 to 100 μm were formed, indicating excellent performance in both cell encapsulation and long-term viability maintenance. This performance, in terms of cell activity and spheroid morphology, was comparable to that of Matrigel, validating the potential of this functionalized hydrogel for application in breast cancer organoid construction [50, 51].

This study leveraged the high-yield expression and efficient purification of ELPs to address challenges in recombinant peptide production, including cost, purification complexity, and batch-to-batch consistency. These findings provide systematic evidence that ELP-SA hydrogels, while preserving the biological functions of natural matrices, overcome clinical translation bottlenecks associated with animal-derived materials, such as batch-to-batch variability and potential immunogenicity.

However, several limitations remain to be addressed in future work. First, the study was primarily based on *in vitro* cell models, lacking long-term *in vivo* evaluation; therefore, the degradation behavior, immune response, and regenerative efficacy of the material under physiological conditions cannot yet be fully determined. Subsequent studies in full-thickness skin defects and chronic wounds (e.g. diabetic models) are recommended to validate long-term therapeutic efficacy and safety. Second, the degradation kinetics and time-dependent mechanical properties of the hydrogels were not systematically assessed, including mass loss in physiological buffer and enzymatic conditions, degradation product analysis, and compression/shear mechanics over time, which are crucial for predicting functional durability *in vivo*. Third, the cell types employed in this study were limited, as HUVECs were primarily used; further studies involving human-derived macrophages, skin fibroblasts, primary endothelial cells, and more physiologically relevant 3D co-culture or tissue explant models are needed to improve biological relevance. Fourth, concentration-dependent experiments revealed that peptide aggregation and precipitation may occur at high concentrations, reducing the effective bioavailable dose. This suggests that further drug release kinetics and *in vivo* pharmacokinetic studies are necessary to optimize sustained-release strategies. Fifth, at the mechanistic level, although preliminary evidence of integrin recognition and pro-angiogenic activity was provided, downstream signaling pathways have not been thoroughly validated using inhibitor-based or gene-silencing approaches. Despite these limitations, the present findings provide a solid foundation, while highlighting the need for future studies focusing on long-term *in vivo* validation, degradation/release kinetics, mechanistic elucidation, and process optimization to accelerate clinical translation of this material.

## Conclusion

This study systematically investigated the design, fabrication, and functional evaluation of calcium-induced composite hydrogels composed of ELPs and SA. Four recombinant ELP variants (ELP-Vs) with distinct functionalities: crosslinking capacity, cell adhesion, pro-angiogenic activity, and anti-inflammatory potential were successfully constructed via genetic engineering. An efficient and high-purity ITC purification process was established, significantly simplifying the protein purification workflow [52]. Subsequently, a Ca^2+^-triggered dual-network ELP-SA hydrogel was developed, with optimized gelation formulation and crosslinking conditions, resulting in favorable mechanical strength and tunable microstructure suitable for soft tissue engineering applications.

Functional evaluation revealed excellent biocompatibility and cell-supporting capacity. ELPR and ELPS peptides significantly enhanced cell adhesion and angiogenesis, whereas ELPL-based hydrogels exhibited broad-spectrum antibacterial effects and promoted M2-type macrophage polarization, suggesting their potential for anti-inflammatory therapy. The optimized composite hydrogel overcomes the biological performance limitations of conventional SA hydrogels, supporting excellent cell viability and spheroid formation in 3D culture of MCF-7 cells. Its biological performance is comparable to that of commercial Matrigel, demonstrating broad application potential.

In conclusion, this study established a structurally tunable and functionally versatile ELP-SA composite hydrogel system, providing a novel scaffold material for tissue engineering and regenerative medicine. Future studies should focus on enhancing its biodegradability, immunomodulatory mechanisms, biological adaptability, and *in vivo* applicability to advance its translational potential for clinical use.

## Supplementary Material

rbaf120_Supplementary_Data

## Data Availability

All the data needed to evaluate the conclusions of the study are presented in this paper. Additional data related to this study were requested from the authors. The datasets presented in this study can be found in the online repositories.
