# Supplementary material for: Design and evaluation of ionically crosslinked multifunctional ELP-SA composite hydrogels for 3D cell culture
Source: Regen Biomater. 2025 Nov 24;12:rbaf120. doi: 10.1093/rb/rbaf120 (PMC12721863; doi:10.1093/rb/rbaf120)
Supplement: rbaf120_Supplementary_Data [file rbaf120_supplementary_data.docx]

**Supplementary Materials**

**Design and Evaluation of Ionically Crosslinked Multifunctional ELP-SA Composite Hydrogels for Biomedical Applications**

*Yiying Chen^1^, Yangmin Wang^1^, Yuxi Li^1^ Xingyang Chen^2^, Wenyun Zheng^2*^, Tianwen Wang^3*^, Hao Jia^4^，Xingyuan Ma^1*^*

*1. School of Biotechnology and State Key Laboratory of Bioreactor engineering, East China University of Science and Technology, Shanghai 200237, China*

*2. School of Pharmacy, Shanghai Key Laboratory of New Drug Design, East China University of Science and Technology, Shanghai 200237, China*

*3. College of Biological and Food Engineering Anhui Polytechnic University, Wuhu 241000, China.*

*4.Shanghai Key Laboratory for Tumor Microenvironment and Inflammation, Department of Biochemistry & Molecular Cellular Biology, Shanghai Jiaotong University School of Medicine, Shanghai 200025, China*

*^#^These authors contributed equally to this work.*

**Correspondence:*

*Xingyuan Ma*

[*maxy@ecust.edu.cn*](mailto:maxy@ecust.edu.cn)

*Tianwen Wang*

*wangtw@mail.ahpu.edu.cn*

*Wenyun Zheng*

[*zwy@ecust.edu.edu.cn*](mailto:zwy@ecust.edu.edu.cn)

**Table. S1.** Comparative analysis of key indicators for the preparation of ELP-Vs solutions by three downstream processes

|  | ELP-Vs | ELPK | ELPR | ELPS | ELPL |
| --- | --- | --- | --- | --- | --- |
|  | Yield (mg/L) | 36.17±2.54 | 34.72±3.29 | 44.72±1.03 | 30.82±1.89 |
| Ni | 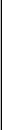Recovery% | 51.501±1.213 | 57.392±0.871 | 48.379±1.192 | 20.972±2.303 |
|  | Purity% | 94.364±0.832 | 95.688±0.544 | 92.671±0.058 | 87.468±0.227 |
| (NH_3_)_2_SO_4_-ITC | Salting-Out  Efficiency% | 83.619±0.132 | 83.954±0.110 | 86.975±0.086 | 83.984±2.164 |
|  | 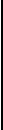Recovery% | 66.792±3.173 | 77.742±2.212 | 84.201±0.940 | 48.390±1.189 |
|  | Purity% | 95.072±0.951 | 96.768±0.483 | 96.422±2.203 | 92.388±1.073 |
| NaCl-ITC | 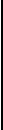Salting-Out  Efficiency% | 92.907±0.318 | 83.342±0.092 | 88.876±0.048 | 92.312±0.213 |
|  | Recovery% | 80.041±2.203 | 74.866±2.129 | 84.622±1.174 | 54.809±3.124 |
|  | Purity% | 95.383±0.952 | 97.682±1.221 | 98.379±1.392 | 97.504±2.307 |


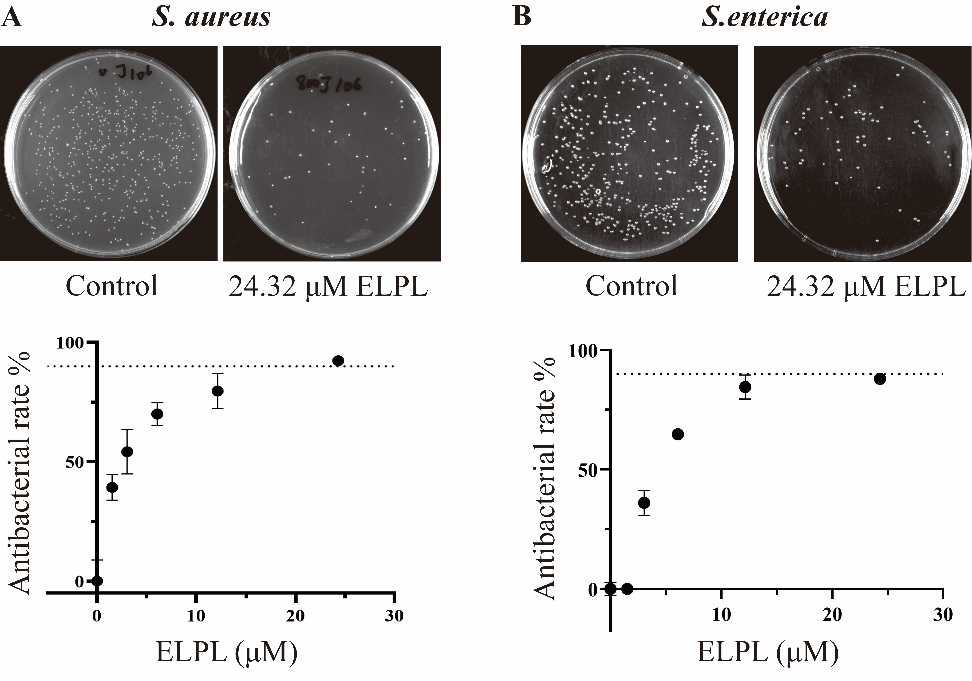


**Figure S1.** Antibacterial effects of ELPL solution against *S. aureus* and *S.enterica*
